# Comparison of the Prostate Imaging Reporting and Data System (PI-RADS) Version 1 and 2 in a Cohort of 245 Patients with Histopathological Reference and Long-Term Follow-Up

**DOI:** 10.5334/jbr-btr.1147

**Published:** 2016-11-24

**Authors:** Pieter De Visschere, Eva Pattyn, Piet Ost, Tom Claeys, Nicolaas Lumen, Geert Villeirs

**Affiliations:** 1 Ghent University Hospital, BE

**Keywords:** Prostate neoplasms, Magnetic Resonance Imaging, Diffusion Weighted Imaging, MR spectroscopic imaging, Prostate cancer

## Abstract

**Objective:**

To compare the performance of PI-RADSv2 with PI-RADSv1 in patients with elevated PSA before biopsy.

**Methods:**

245 patients with elevated PSA underwent mpMRI before biopsy between May 2011 and December 2014 at 3.0 Tesla without endorectal coil. Patients underwent transrectal ultrasound-guided systematic 12-core biopsy followed by radical prostatectomy (N = 68), radiation therapy (N = 91) or clinical follow-up for at least two years (N = 86). All exams were scored on a per-patient basis according to PI-RADSv1 and PI-RADSv2. ClinsigPC was defined as Gleason score ≥7 (including 3+4 with prominent but not predominant Gleason 4 component), and/or tumour volume of ≥0.5cc, and/or tumour stage ≥T3a.

**Results:**

In 144 patients (58.8%) a ClinsigPC was found within two years after mpMRI. The PI-RADSv1 and PI-RADSv2 overall assessment scores were significantly higher (P < 0.001) in patients with ClinsigPC as compared to patients without ClinsigPC. ROC analysis showed an area under the curve of 0.82 (CI 0.76–0.87) for PI-RADSv1 and 0.79 (CI 0.73–0.85) for PI-RADSv2 (P: NS). A threshold score of 3 exhibited sensitivities of 88.2% and 79.2% (P = 0.001) and specificities of 64.4% and 67.3% (P: NS) with PI-RADSv1 and PI-RADSv2, respectively.

**Conclusions:**

The mpMRI scoring systems PI-RADSv1 and PI-RADSv2 yield similar accuracy to detect ClinsigPC in patients with elevated PSA, although clinicians should be aware that when an overall assessment score of 3 is used as a threshold for a positive mpMRI, PI-RADSv2 has lower sensitivity than PI-RADSv1. Nevertheless, PI-RADSv2 is preferable over PI-RADSv1 because it has the advantage of providing well-defined instructions on how to determine the overall assessment category.

## Introduction

Prostate cancer (PC) is the most frequent non-cutaneous tumor in men but up to 40% of PC will never cause symptoms or death and should be considered clinically insignificant [1]. Multiparametric magnetic resonance imaging (mpMRI) has become an important imaging technique in the assessment of patients with known or suspected PC to detect or rule out clinically significant disease. In a mpMRI morphological T2-weighted imaging (T2-WI) are supplemented with diffusion-weighted imaging (DWI), dynamic contrast-enhanced imaging (DCE) and/or magnetic resonance spectroscopic imaging (MRSI) [2]. Reporting prostate mpMRI may be complex and prone to subjective interpretation, therefore the European Society of Urogenital Radiology (ESUR) published in 2012 guidelines and proposed the Prostate Imaging Reporting and Data System version 1 (PI-RADSv1) [2]. The PI-RADSv1 scoring system involves assignment of separate scores to each of the modalities and provides explicit verbal descriptions on how to generate them. Each exam is assigned with an overall assessment score ranging from 1 (indicating that clinically significant cancer is highly unlikely to be present) to 5 (indicating that clinically significant cancer is highly likely to be present) to communicate the conclusion to the referring clinician. This overall assessment category score is based on a subjective radiologist’s impression weighting the results of the single modalities. Whenever the results of the single scores are incoherent, one of the modalities is preferred over the others to generate the overall assessment score. PI-RADSv1 thus lacks a consistent instruction on how to calculate the overall assessment score [2,3,4]. This shortcoming has resulted in the creation of a modified version in 2015, named PI-RADS version 2 (PI-RADSv2) which was also adopted by the American College of Radiology (ACR) [5,6]. In PI-RADSv2 two dominant modalities have been chosen, namely DWI for the peripheral zone (PZ) and T2-WI for the transition zone (TZ) and well-defined instructions have been provided on how to determine the overall assessment score.

In the current study we aimed to compare the diagnostic performance of PI-RADSv2 with PI-RADSv1 to detect clinically significant PC in patients with elevated PSA. We hypothesized that they were similar and in that case the previously established validation data of prostate mpMRI that were obtained using PI-RADSv1 might still be valid in the future for mpMRI that are performed using PI-RADSv2.

## Materials and Methods

### Patients

All patients with elevated PSA who underwent prostate mpMRI before biopsy at our institution between May 2011 and December 2014 were eligible for this study. Patient files were retrospectively explored to collect histopathological and clinical follow-up data as reference. Patients who had been treated for PC or had previous transurethral resection of the TZ as treatment of benign prostatic hyperplasia were excluded. Two-hundred and forty-five patients had adequate histopathological and clinical follow-up data for the purposes of this study. Patients were included if they underwent a systematic 12-core TRUS-guided prostate biopsy followed by radical prostatectomy (N = 68) or primary radiation therapy (N = 91) within two years after the mpMRI. In all patients the presence of PC was initially established with a systematic 12-core TRUS-guided prostate biopsy. The biopsy cores were collected in eight separate containers: left and right prostate base, apex, midprostate and transition zone. In patients treated by radical prostatectomy the histopathological conclusion of the prostatectomy specimen was used as the reference for the study instead of the biopsy result in case of discordance (upgrading or downgrading). In the patients treated with primary radiation therapy the diagnosis relied solely on the prostate biopsy. Patients not undergoing active treatment were included if they were followed up for at least two years with repetitive PSA measurements, digital rectal examination, TRUS-guided systematic 12-core prostate biopsies and/or repeat mpMRI at the discretion of the referring urologist (N = 86). In the latter group, 13 patients with a PC were followed in an active surveillance program. The remaining 73 patients had no evidence of tumor at initial biopsy and were without evidence of PC two years after the mpMRI. A patient was considered free of PC if for example the PSA value normalized spontaneously in the months after the MRI, or if repetitive TRUS biopsies were negative, or if a new mpMRI still showed no suspicious lesions. PC was considered clinically significant (ClinsigPC) if the Gleason score was ≥7 (including 3+4 with prominent but not predominant Gleason 4 component), and/or PC volume ≥0.5cc, and/or extraprostatic extension, as proposed in PI-RADSv2 (5). The study was approved by our hospital’s Ethics Committee (EC 2011/495 with amendment dd. 18–11–2015).

### mpMRI technique

The mpMRI consisted of T2-WI, DWI, DCE and MRSI and was performed at 3.0 Tesla without endorectal coil (Magnetom Trio, Siemens Medical Systems, Erlangen, Germany). The acquisition parameters used for the study are presented in supplemental Table 1.

**Table 1:** Histopathological findings and clinical follow-up data.

Histopathology and clinical diagnosis based on biopsy followed by:	N	Gleason score				T-stage				clinsigPC	
		NC	LG	IG	HG	T1	T2	T3	T4	No	Yes

Radical prostatectomy	68	1	5	33	29	0	44	23	0	3	65
Radiation therapy	91	0	18	26	47	28	40	20	3	14	77
Active surveillance > 2 years	13	0	11	2	0	13	0	0	0	11	2
>2 years of cancer free follow-up	73	73	/	/	/	/	/	/	/	73	0
Total	245	74	34	61	76	41	84	43	3	101	144

NC: No prostate cancer. LG: Low Grade Prostate Cancer, defined as prostate cancer with Gleason score 3+3 or lower. IG: Intermediate Grade Prostate Cancer, defined as prostate cancer with Gleason score 3+4. HG: High Grade Prostate Cancer, defined as any prostate cancer with primary Gleason grade 4 or any Gleason grade 5 (including tertiary patterns). ClinsigPC: Gleason score ≥7 (including 3+4 with prominent but not predominant Gleason 4 component), and/or volume ≥0.5cc, and/or extraprostatic extension.

All exams were anonymized and in January 2015 all mpMRI were evaluated according to the PI-RADSv1 and PI-RADSv2 scoring systems on a dedicated workstation (Leonardo, Siemens Medical Systems, Erlangen, Germany) by a single reader with 10 years of experience in prostate mpMRI. The PI-RADSv1 and PI-RADSv2 scores were assigned strictly following the descriptions of the different parameters in the respective guidelines. They were assigned during the same reading session because we wanted to avoid that a lesion was noted by the reader when scoring PI-RADSv1 but overlooked when scoring PI-RADSv2 in a separate session. This would cause differences between the scores that were not caused by the differences in the guidelines lexicon but caused by the reader.

The image interpretation included an overall assessment category based on subjective weighting of the scores of the single modalities in PI-RADSv1 and based solely on the dominant modalities for PI-RADSv2 [2,5]. We did not use the sum score for PI-RADSv1 as has been applied by several researchers because this method was not mentioned nor recommended in the PI-RADSv1 guidelines. MRSI score was not taken into account for scoring PI-RADS v2.

### Statistical analysis

For statistical analysis, relationships between PI-RADSv1 and PI-RADSv2 scores and the presence of ClinsigPC on a per-patient basis were assessed by the Mann-Whitney U-test. The balance between sensitivity and specificity for different thresholds was analyzed by receiver operating characteristic curves (ROC) and was conducted for the overall assessment categories and for the single mpMRI modality scores using the presence of ClinsigPC on a per-patient basis as the gold standard. The PI-RADSv1 and PI-RADSv2 overall assessment categories were compared with the McNemar test. For the evaluation of the performance of PI-RADSv1 and PI-RADSv2 the scores were dichotomized in ‘negative’ in case of an overall assessment score of 1 or 2 and ‘positive’ in case of a score 3, 4 or 5. The level of significance was set at 0.05. For all statistical analyses SPSS for Windows version 22.0 (SPSS, Chicago, Ill) was used.

## Results

In 144 patients (58.8%), a ClinsigPC was detected within two years following mpMRI.

Histopathological and clinical follow-up data are demonstrated in Table 1. The median age of the patients was 66 years old (range 44–85 years), PSA 9 μg/l (range 1.4–935.5 μg/l) and prostate volume 49.3 cc (range 19.8–201.0 cc). The overall assessment scores were significantly higher (*P* < 0.001) in patients with ClinsigPC (median: 4, 25th percentile: 3, 75th percentile: 5) as compared to patients without ClinsigPC, (median: 2, 25th percentile: 2, 75th percentile 3) in both PI-RADSv1 and PI-RADSv2 indicating that they are both useful scoring systems to provide relevant risk stratification of ClinsigPC.

In PI-RADSv1 more patients were assigned an overall assessment score 3 (17.6% vs 10.2%) (*P* = 0.005) and fewer a score 2 (28.6% vs 35.1%) (*P* = 0.02) (Table 2) although the positive predictive values of the overall assessment scores were not statistically significantly different from PI-RADSv2. An increase in probability of ClinsigPC was observed with increasing overall assessment score in both PI-RADSv1 and PI-RADSv2. An overall assessment score 5 had a positive predictive value for the presence of ClinsigPC of 90.4% when scored with PI-RADSv1 and 93.0 % when scored with PI-RADSv2 (*P* = NS).

**Figure 1:**
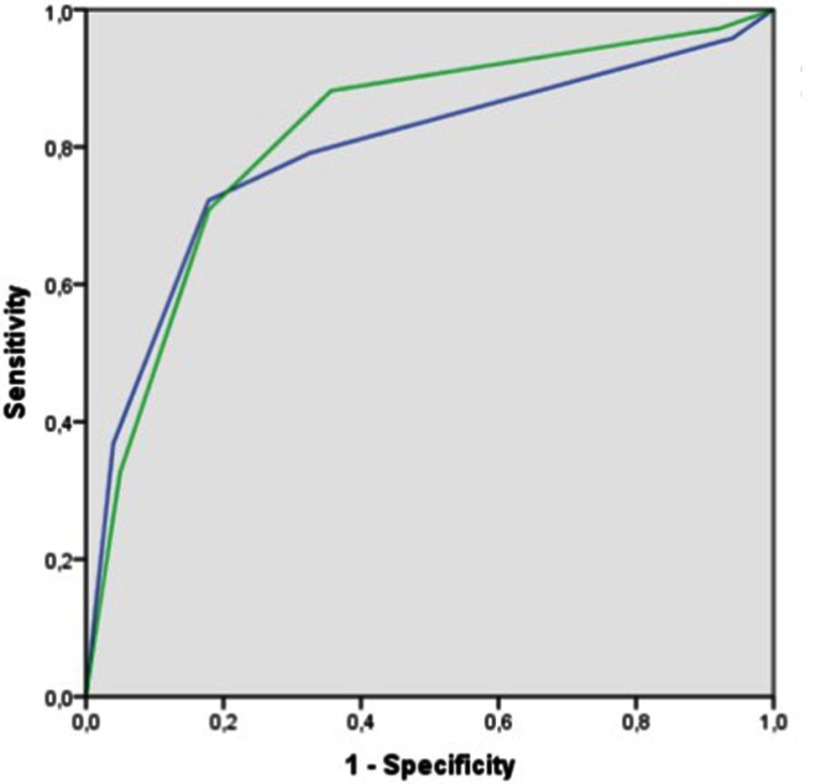
ROC curves of the overall assessment scores of PI-RADSv1 (green line, AUC 0.82 [95% CI 0.76–0.87]) and PI-RADSv2 (blue line, AUC 0.79 [CI 0.73–0.85]) using the presence of ClinsigPC on a per-patient basis as the reference.

The ROC analysis using the presence of ClinsigPC on a per patient base (Figure 1) showed an area under the curve (AUC) of 0.82 [95% CI, 0.76–0.87] for PI-RADSv1 and 0.79 [95% CI, 0.73–0.85] for PI-RADSv2 (*P* = NS) indicating clinically relevant predictive characteristics for both scoring systems. Evaluation of the separate mpMRI modalities showed no significant differences in AUCs between PI-RADSv1 and PI-RADSv2 respectively for the scores of T2-WI in the PZ (0.78), T2-WI in the TZ (0.52 vs 0.53), DWI (0.82 vs 0.81) and DCE (0.81 vs 0.79) (*P* = NS) (Table 3).

**Table 2:** Overall assessment scores and probability of clinically significant prostate cancer.

Overall assessment score	Frequency of assessed score			Probability of ClinsigPC		
	PI-RADSv1	PI-RADsv2	*P*-value	PI-RADSv1	PI-RADSv2	*P*-value

1	4.9% (12/245)	4.9% (12/245)	NS	33.3% (4/12)	50.0% (6/12)	NS
2	28.6% (70/245)	35.1% (86/245)	0.02	18.6% (13/70)	27.9% (24/86)	NS
3	17.6% (43/245)	10.2% (25/245)	0.005	58.1% (25/43)	40.0% (10/25)	NS
4	27.8% (68/245)	26.5% (65/245)	NS	80.9% (55/68)	78.5% (51/65)	NS
5	21.2% (52/245)	23.3% (57/245)	NS	90.4% (47/52)	93.0% (53/57)	NS

**Table 3:** AUC values of the ROC analysis for the overall assessment score and the different individual modalities.

mpMRI modality	Area under the curve (95% confidence interval)		*P*-value
	PI-RADSv1	PI-RADSv2	

Overall assessment score	0.82 (0.76-0.87)	0.79 (0.73-0.85)	NS
T2-WI in PZ	0.78 (0.72-0.84)	0.78 (0.72-0.84)	NS
T2-WI in TZ	0.52 (0.45-0.60)	0.53 (0.45-0.60)	NS
DWI	0.82 (0.77-0.88)	0.81 (0.76-0.87)	NS
DCE	0.81 (0.75-0.87)	0.76 (0.70-0.83)	NS
MRSI	0,65 (0.58-0.72)	/	/

When an overall assessment score of 4 was used as a threshold for a positive mpMRI, the performance of PI-RADSv1 and PI-RADSv2 was not significantly different, with accuracy of 75.5% and 76.3%, respectively (*P* = NS), sensitivity of 70.8% and 72.2% (*P* = NS) and specificity of both 82.2% (*P* = NS). When an overall assessment score of 3 was used as a threshold for a positive mpMRI, the accuracy of PI-RADSv1 and PI-RADSv2 was not significantly different (78.4% vs 74.3%) (*P* = NS), but PI-RADSv1 showed significantly higher sensitivity than PI-RADSv2 (88.2% vs 79.2%) (*P* = 0.001) for similar specificity (64.4% vs 67.3%) (*P* = NS).

The overall assessment scores of PI-RADSv1 and PI-RADSv2 were identical in 64.9% (159/245). With an overall assessment score of 3 as a threshold for a positive mpMRI, the dichotomized scores were concordant in 87.8% (215/245). In 46.1% (113/245) PI-RADSv1 and PI-RADSv2 were both true positive, in 24.1% (59/245) they were both true negative. In 27 patients (11.0%) PI-RADSv1 and PI-RADSv2 were both considered ‘false positive’ according to the definition of ClinsigPC that was used although actually in half of these cases (55.5%, 15/27) a PC was present, but considered clinically insignificant (a small Gleason 3+3 PC in 12 patients and a small Gleason 3+4 PC in 3 patients). In 16 patients (6.5%) a ClinsigPC was missed on both PI-RADSv1 and PI-RADSv2, but only a minority (5/16, 31.2%) were Gleason 4+3 PC or higher.

**Figure 2:**
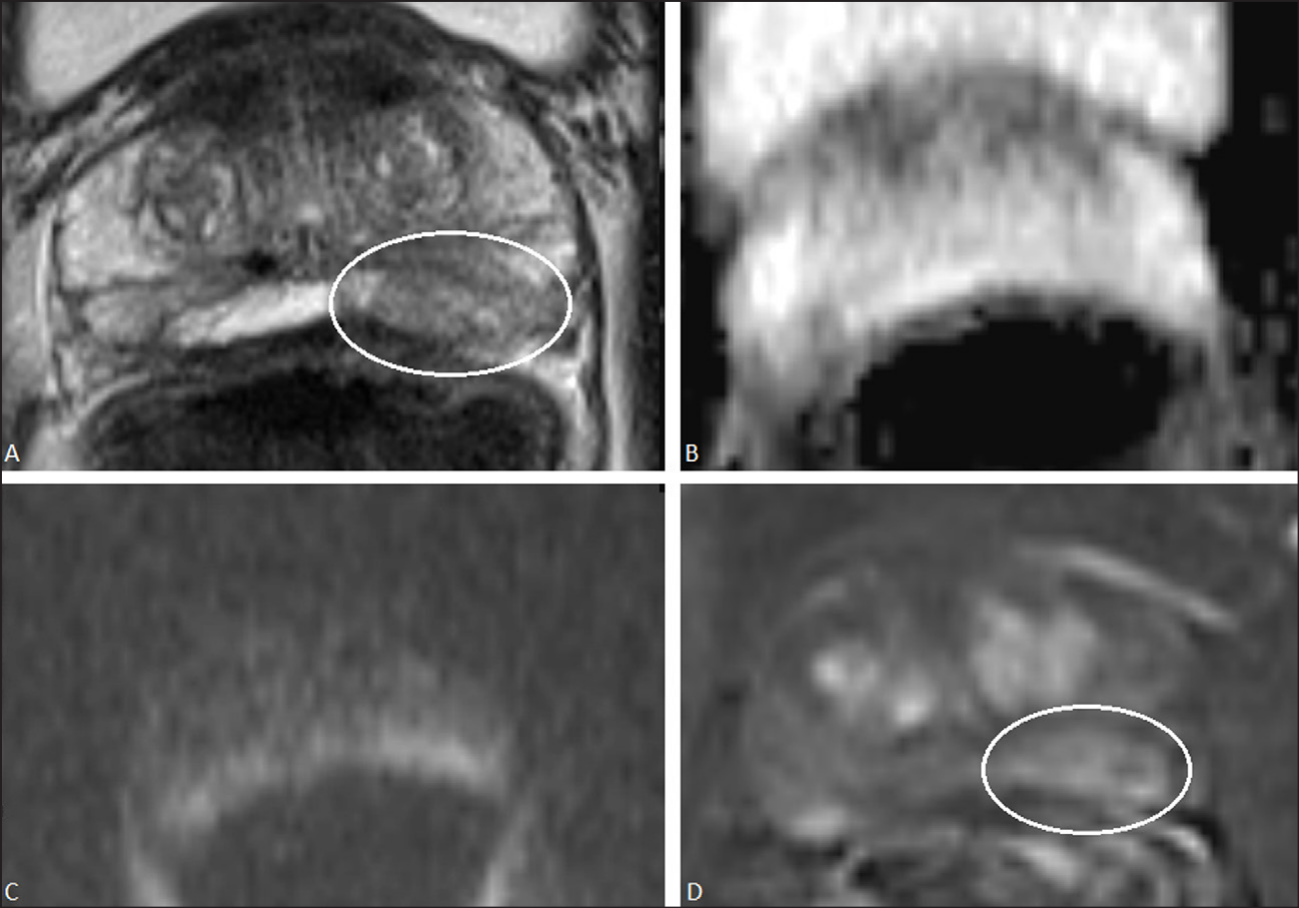
A 63-year-old man with a PSA of 6.3 μg/L. On T2-WI **(a)** in the PZ an ill-defined low signal intensity focus is present posteriorly on the left side in the prostate base (white oval) (PI-RADSv1 score 4; PI-RADSv2 score 4). On DWI the signal intensity on ADC is high **(b)** and on high-b-value image isointense/mildly hyperintense **(c)** (PI-RADSv1 score 1; PI-RADSv2 score 1). On DCE (4) the area shows strong contrast enhancement (white oval) (PI-RADSv1 score 4; PI-RADSv2 negative). With the PI-RADSv1 scoring system this patient was assigned an overall assessment score 4, based on a subjective impression of the findings on T2-WI and DCE. With the PI-RADSv2 scoring system, this patient was assigned an overall assessment score of 1 since DWI is the dominant modality in the PZ. A prostate biopsy followed by radical prostatectomy in this patient showed however a Gleason 3+4 prostate cancer on the left side in the PZ of the prostate base.

**Figure 3:**
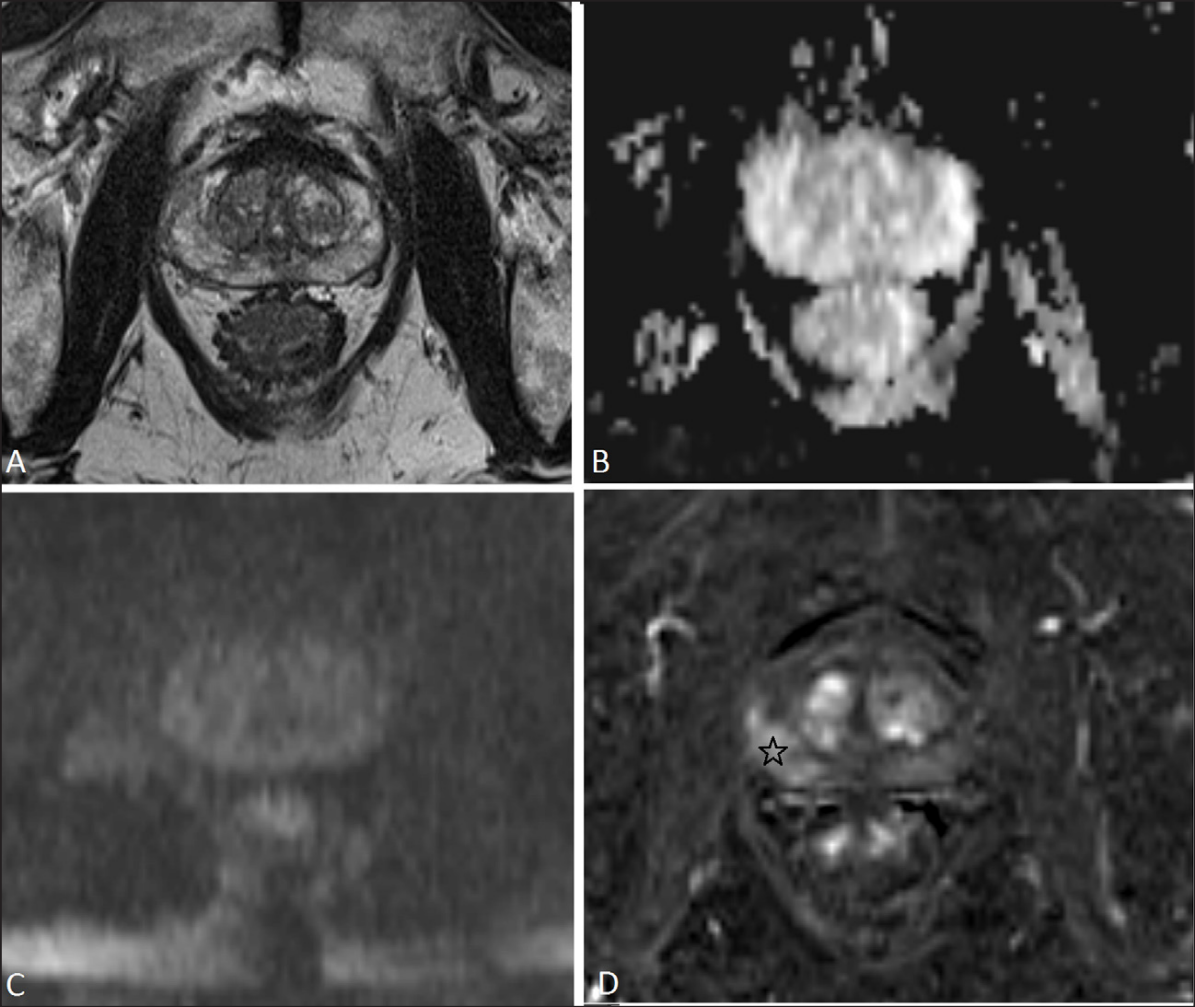
A 66-year-old man with PSA 9.8μg/L. On T2-WI **(a)** the PZ shows diffuse mild hypointensity, with indistinct margin (PI-RADSv1 score3; PI-RADSv2 score 3). On DWI the PZ shows high ADC values **(b)** and is isointense on high-b-value image **(c)** (PI-RADSv1 score 1; PI-RADSv2 score 1). On DCE there is a focal enhancing lesion posterolateral on the right side in the PZ (black star) (PI-RADSv1 score 4; PI-RADSv2 positive). With the PI-RADSv1 scoring system, this patient was assigned an overall assessment score 4, based on a subjective overall impression of the findings in all modalities. With the PI-RADSv2 scoring system this patient was assigned an overall assessment score 1 because DWI was scored 1 and this is the dominant modality in PZ. Prostate biopsy followed by radical prostatectomy in this patient showed a Gleason 3+4 prostate cancer on the right side in the PZ.

**Figure 4:**
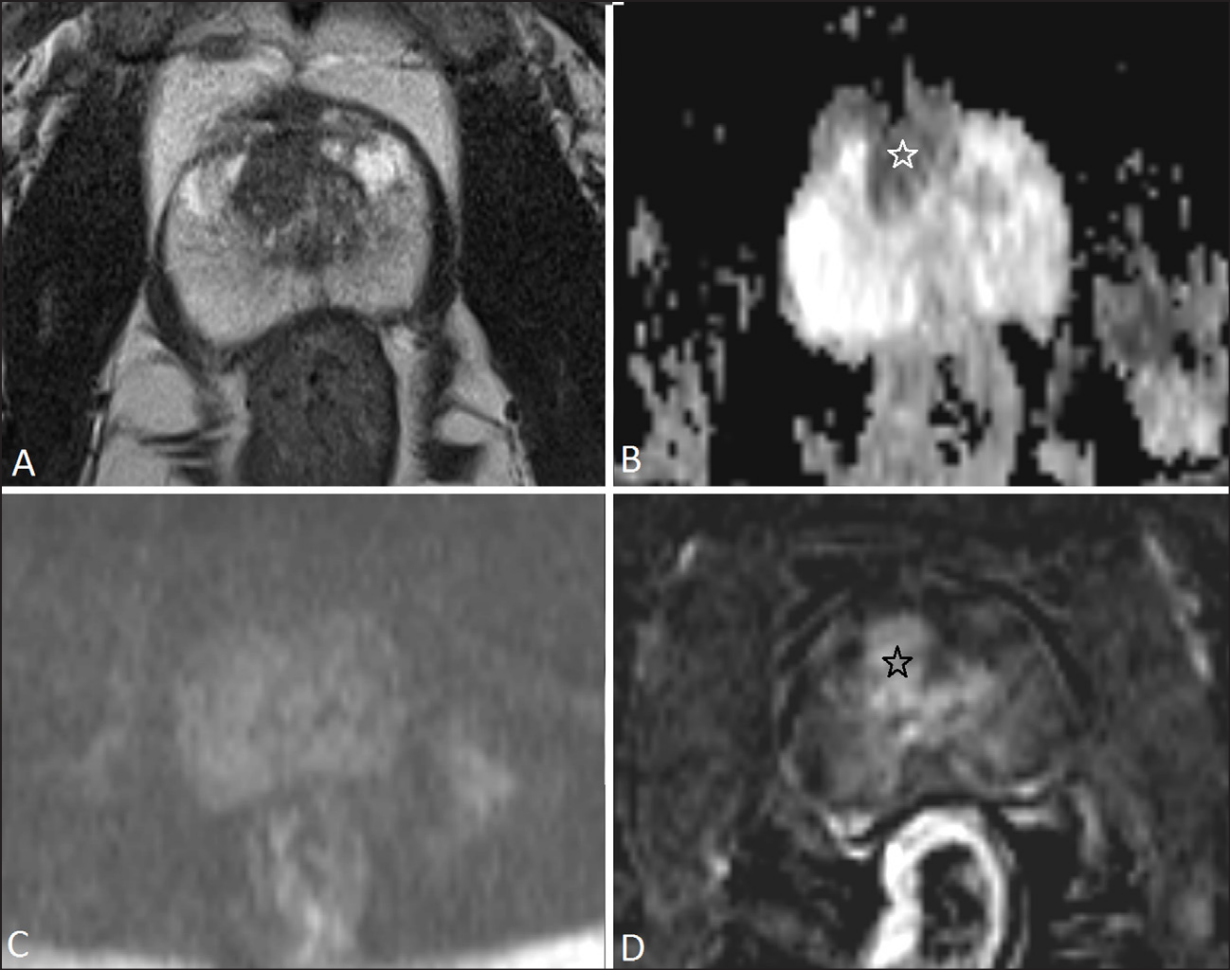
A 56-year-old man with PSA of 11.2μg/L. On T2-WI **(a)** the PZ shows a homogenous high signal intensity (PI-RADSv1 score 1; PI-RADSv2 score 1) and in the TZ a well-margined homogenous hypointense area is noted (PI-RADSv1 score 2; PI-RADSv2 score 2). On DWI this area in the TZ shows low ADC value **(b)** (white star) and is isointense on high-b-value image **(c)** (PI-RADSv1 score 4; PI-RADSv2 score 4). On DCE this area shows strong contrast enhancement (black star) (PI-RADSv1 score 4; PI-RADSv2 positive). With the PI-RADSv1 scoring system this patient was assigned an overall assessment score 4, based on a subjective overall impression of the findings in all modalities. With the PI-RADSv2 scoring system, T2-WI in the TZ was scored 2, and since T2-WI is the dominant modality in the TZ the overall assessment score was also 2. Prostate biopsy followed by radical prostatectomy in this patient showed a Gleason 3+4 prostate cancer in the TZ.

With an overall assessment score of 3 as a threshold for a positive mpMRI, a discrepancy between the PI-RADSv1 and PI-RADSv2 overall assessment scores occurred in 12.2% (30/245) (Table 4). The main cause of this discrepancy (46.7%, 14/30) were focal or diffuse suspicious findings on T2-WI in the PZ with normal findings on DWI. They were scored positive with PI-RADSv1 but negative with PI-RADSv2 and in 71.4% (10/14) a ClinsigPC was present (Figure 2). Another cause (26.7%, 8/30) of discrepancy occurred in patients with focal suspicious contrast enhancement on DCE but with normal findings on all the other modalities (Figure 3). They were scored positive with PI-RADSv1 but negative with PI-RADSv2 and in 37.5% (3/8) a ClinsigPC was present. A third cause (23.3%, 7/30) of discrepancy between PI-RADSv1 and PI-RADSv2 occurred when there was a suspicious lesion on T2-WI and DWI but without focal contrast enhancement on DCE and normal metabolite concentrations on MRSI. They were scored negative with PI-RADSv1 but positive with PI-RADSv2 although a ClinsigPC was present in only 14.3% (1/7). An additional case of discrepancy was caused by a suspicious nodule in the TZ on DWI with contrast enhancement but with normal morphology on T2-WI, therefore scored positive with PI-RADSv1 but negative with PI-RADSv2 although a ClinsigPC was present (Figure 4).

**Table 4:** Discrepancies between PI-RADSv1 and PI-RADSv2.

PI-RADSv1 and PI-RADSv2 discrepancy^1^	12,2% (30/245)	Overall assessment scores			Histopathological findings	Reason discrepancy on mpMRI
		N	PI-RADSv1	PI-RADSv2		

Missed ClinsigPC on PI-RADSv1 but true positive on PI-RADSv2	3,3% (1/30)	100% (1/1)	2	4	The ClinsigPC was a large Gleason 3+3 PC	T2-WI and DWI suspicious, but DCE and MRSI normal
True negative on PI-RADSv1 but false positive on PI-RADSv2	20,0% (6/30)	83,3% (5/6)	2	3	No ClinsigPC: no cancer in 4 cases, but a small Gleason 3+3 PC in 1 case	In all cases T2-WI and DWI suspicious, but DCE and MRSI normal
		16,7% (1/6)	2	4	No ClinsigPC although there was a very small Gleason 3+4 PC	
False positive on PI-RADSv1 but true negative on PI-RADSv2	30,0% (9/30)	11,1% (1/9)	3	1	No cancer	In 5 cases DCE suspicious but all other modalities normal and in 4 cases T2-WI suspicious but DWI normal in PZ
		77,8% (7/9)	3	2	No ClinsigPC although there was 1 case of small Gleason 3+3 PC, 2 cases of HGPIN & BCH and 1 case of ASAP	
		11,1% (1/9)	4	2	No cancer	
True positive on PI-RADsv1 but missed ClinsigPC on PI-RADSv2	46,7% (14/30)	7,1% (1/14)	3	1	The ClinsigPC was a Gleason 4+3 PC	In 3 cases DCE suspicious but all other modalities normal, in 10 cases T2-WI suspicious but DWI normal in PZ and in 1 case only DWI suspicious in TZ
		78,6% (11/14)	3	2	The ClinsigPC were 1 Gleason 3+3 pT2c, 6 Gleason 3+4 PC and 4 Gleason 4+3 PC or higher	
		7,1% (1/14)	4	2	The ClinsigPC was a Gleason 3+4 PC	
		7,1% (1/14)	5	2	The ClinsigPC was a Gleason 3+4 PC	

^1^After dichotomization with considering an overall assessment score 1 and 2 as ‘negative’ and 3, 4 or 5 as ‘positive’ mpMRI.

## Discussion

The present study demonstrated that in patients with elevated PSA both PI-RADSv1 and PI-RADSv2 were valid scoring systems for the risk stratification of ClinsigPC. The ROC analysis in our study exhibited AUC of 0.82 [95% CI, 0.76–0.87] for PI-RADSv1 and 0.79 [95% CI, 0.73–0.85] for PI-RADSv2, which were similar to those reported in literature by Schimmöller [7] (AUC 0.81) and Portalez [8] (AUC 0.86) using PI-RADSv1 and by Muller [9] (0.86 for PZ, 0.87 for TZ) using PI-RADSv2 indicating the ability of both standardized scoring systems to stratify mpMRI findings by cancer suspicion. When an overall assessment score of 4 was used as a cut-off level for a positive mpMRI, the performance of PI-RADSv1 and PI-RADSv2 was not significantly different, but when a threshold of 3 was applied, then the sensitivity of scoring with PI-RADSv2 was lower than with PI-RADSv1. An overall assessment score of 3 indicates that the presence of clinically significant disease is equivocal. This indeterminate mpMRI remains a problem and is currently still an important issue of debate. Nevertheless, in the clinical setting of mpMRI being performed in patients with elevated PSA as additional parameter (next to clinical biomarkers such as PSA level, digital rectal examination and patient’s age) to decide to biopsy or not, an overall assessment score of 3 cannot exclude the presence of a clinsigPC with high enough certainty. Therefore it seems not prudent to omit or postpone the biopsy in these cases, and therefore most clinicians will consider a score 3 as a positive sign to perform a biopsy. The MRI findings are then still useful for targeting the equivocal area. Considering an overall assessment score 3 as a positive mpMRI might lead to some overdiagnosis and clinicians should be aware that the decision to biopsy may then slightly be influenced by the scoring system that is used by the radiologist. It may be recommendable to discuss these cases at multidisciplinary meetings. With an overall assessment score of 3 as a threshold for a positive mpMRI a sensitivity and specificity of 88.2% and 64.4% respectively, were obtained with PI-RADSv1 and 79.2% and 67.3%, with PI-RADSv2. Our findings are consistent with previously published results where sensitivities and specificities of 71–84% and 33–70% for PC of any grade, and of 80–90% and 47–61% for high-grade PC are reported with PI-RADSv1 [8,10,11,12,13,14,15,16,17,18,19,20] and 88% and 71% in the PZ and 85% and 55% in the TZ with PI-RADSv2 [9].

Hamoen et al. [21] performed a meta-analysis of 14 studies evaluating PI-RADSv1 and reported a wide variability in performance with a pooled sensitivity of 74% (95% CI 67%–81%) and a pooled specificity of 80% (95% CI 70%–88%) for studies with detection of any PC as the outcome measure and a pooled sensitivity of 84% (95% CI 76%–89%) and pooled specificity of 75% (95% CI 66%–83%) for studies with detection of ClinsigPC as primary outcome. Rosenkrantz et al. [22] performed an interobserver reproducibility study of PI-RADSv2 and reported a sensitivity of 100% and specificity of 56.5% for detection of Gleason ≥3+4 PC with a score 3 as threshold for a positive mpMRI. These different results as compared to our study are probably related to the reference standard (MRI/US fusion biopsy) that was used and a different clinical setting. It is likely that they have a high proportion of positive mpMRI while we tried to avoid too much (false) positive exams to avoid that otherwise on a per-patient basis no biopsies could be postponed or omitted.

Vargas et al. [23] investigated the impact of pathologic tumor volume on detectability with PI-RADSv2 and reported that PI-RADSv2 correctly identified 94–95% of PC foci of ≥0.5cc of any Gleason score but only 10–26% of PC foci with Gleason ≥4+3 but <0.5cc.

**Figure 5:**
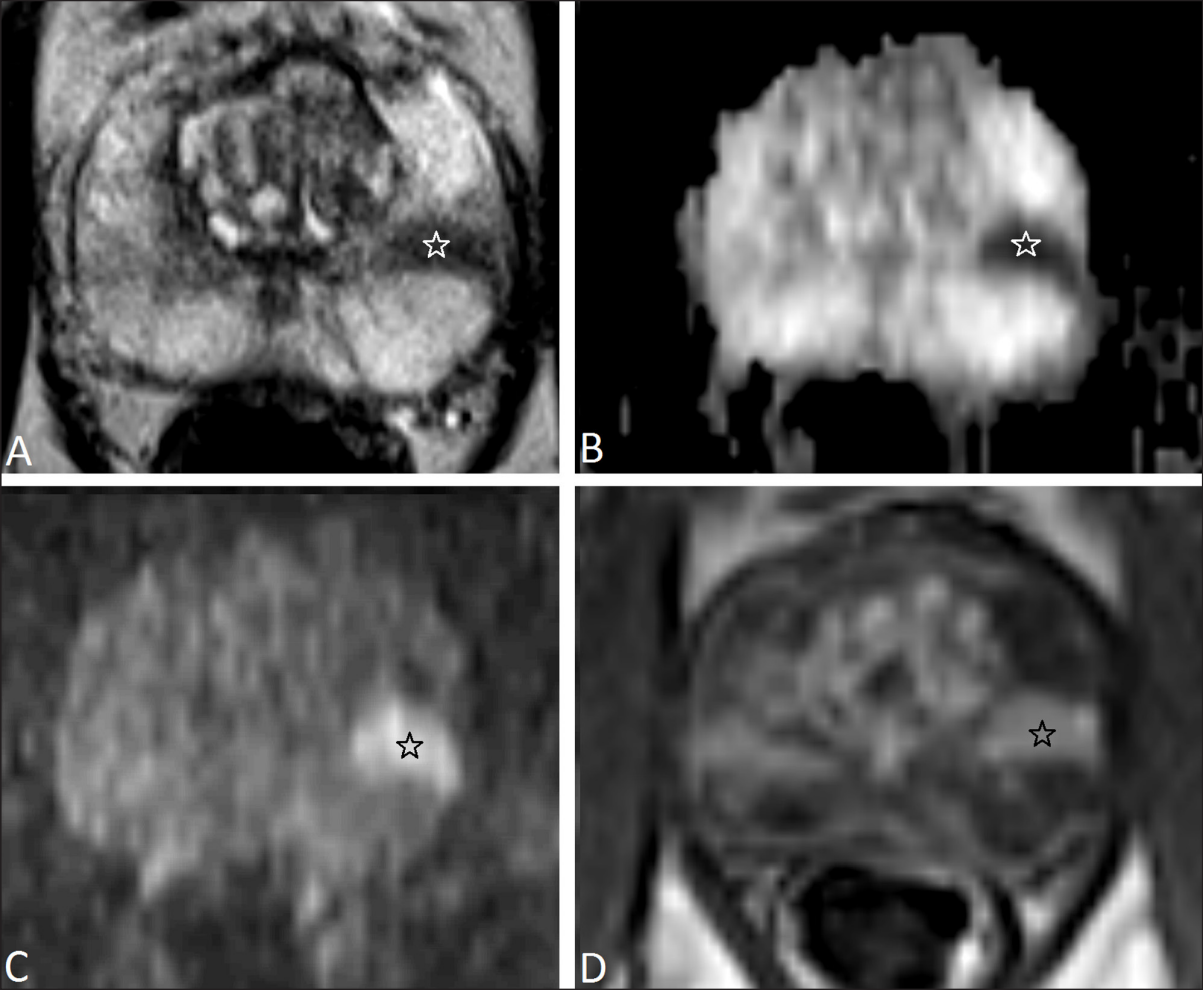
A 63-year-old man with PSA 8.35 μg/L. In the PZ on the left side there is a low signal intensity focus on T2-WI (PI-RADSv1 score 5; PI-RADSv2 score 4) **(a)** (white star) with restricted diffusion (markedly hypointense on ADC **(b)** and hyperintense on high-b-value **(c)**) (PI-RADSv1 score 5, PI-RADSv2 score 4). The size of the lesion is <1,5 cm, it has no broad contact with the prostate capsule and shows no definite extraprostatic extension. On DCE **(d)** it shows strong contrast enhancement (black star) (PI-RADSv1 score 5; PI-RADSv2 positive). The overall assessment score is 5 when using PI-RADSv1 based on a subjective impression of the findings of all modalities and is 4 in PI-RADSv2 based on the DWI-only which should be given a score 4. Despite the different overall assessment category between PI-RADSv1 and PI-RADSv2 the message to the clinician is the same, i.e. targeted prostate biopsy is warranted. Prostate biopsy followed by radical prostatectomy confirmed a PC with Gleason score 3+4 = 7 (with tertiary pattern 5).

The dichotomized overall assessment scores of PI-RADSv1 and PI-RADSv2 in our study were concordant in 87.8%, despite their differences in scoring of the individual modalities and determination of the overall assessment scores. In PI-RADSv1 some verbal descriptors showed imprecise results and were therefore modified when PI-RADSv2 was developed; for example, the scoring of DCE was a complex indicator in PI-RADSv1 because it combined a 3-point score with additional points [2,8,17,18]. The modified verbal descriptions in PI-RADSv2 resulted in our study in different scoring of individual modalities between PI-RADSv1 and PI-RADSv2, but this had in the majority of cases no impact when dichotomizing the scores in positive (score 3, 4 or 5) or negative (score 1 or 2). For example, a very suspicious lesion of <1.5cm in the PZ on DWI was assigned a score 5 in PI-RADSv1 but a score 4 in PI-RADSv2 (Figure 5); both were considered positive and supported the clinician’s decision to biopsy.

In PI-RADSv2 fewer patients were assigned an overall assessment score 3 as compared with PI-RADSv1 (10.2% vs 17.6%), with a shift mainly to an overall assessment score 2 (35.1% vs 28.6%). Discrepancy between the PI-RADSv1 and PI-RADSv2 overall assessment score occurred mainly in patients with focal or diffuse suspicious findings in the PZ on T2-WI but with normal findings on DWI, or in patients with focal suspicious contrast enhancement on DCE but with normal findings on all the other modalities: they were all scored positive with PI-RADSv1 but negative with PI-RADSv2. The AUCs for the T2-WI scores, DWI and DCE scores were not significantly different between PI-RADSv1 and PI-RADSv2, thus the shift in overall assessment scores from 3 to 2 was not caused by differences in the scoring of the individual modalities but was the result of a different way of generating the overall assessment scores.

PI-RADSv1 lacked a consistent instruction on how to weight the scores given to the individual sequences and how to calculate the overall assessment score [1,3]. The complexity and sometimes contradictory findings of the different single modalities resulted in substantial heterogeneity in interpretations of mpMRI in routine clinical practice between institutions and in the analysis and cut-off values used in the PI-RADSv1 scores [21]. Some authors [3,8,18] added the individual scores of each sequence to a total sum score to generate the overall PI-RADSv1 score, based on the assumption that all the modalities had the same diagnostic weight. Variable performances were reported with this arithmetic sum score as compared to the subjective radiologist’s impression approach [3,8,12,20,21,24]. It may seem intuitive to use this method, but it was not mentioned nor recommended in the PI-RADSv1 guidelines. Moreover, the sum score is not necessarily the best option to compare between institutions a PI-RADSv1 score on a 4–20 scale (when MRSI is included) with a PI-RADSv1 score on a 3–15 scale (when MRSI is not included because it is an optional technique) or with a PI-RADSv2 on a 1–5 scale.

PI-RADSv2 has overcome this problem by assigning strict criteria on how to determine the overall assessment category. It was hoped that this would make the interpretation of the PI-RADS score easier, especially for less experienced readers, but Muller et al. [9] reported that PI-RADSv2 had only moderate level of interobserver agreement for readers of varying experience, similar as for PI-RADSv1 [25].

In summary, PI-RADSv1 and PI-RADSv2 yield similar accuracy although a reduction in sensitivity was noted when using PI-RADSv2 with an overall assessment score of 3 as a threshold. Nevertheless, the advantage of PI-RADSv2 to provide well-defined instructions on how to determine the overall assessment category is preferable over PI-RADSv1 with its subjective overall impression method. Further research is necessary to improve the scoring system, and we would like to suggest some modifications that may be taken into account when working towards a PI-RADS version 3. In our study we noted a shift from overall assessment score 3 with PI-RADSv1 to score 2 when using PI-RADSv2, not caused by the assessment of the individual modalities but due to the different way of generating the overall assessment category. The majority of these discrepancies were caused by a suspicious lesion in the PZ on T2-WI but with normal DWI, scored positive with PI-RADSv1 but negative with PI-RADSv2 although a ClinsigPC was actually present in about 60%. Therefore, we suggest that the rules for determination of the overall assessment score might be adapted or clarified; for example, a weighting factor for each modality could be included in order to make it possible that a very suspicious lesion in T2-WI in the PZ could overrule a negative DWI.

There are some limitations to our study. Firstly, we did not have step-section histopathology of radical prostatectomy specimens as gold standard in all patients. Studies using only RP specimens as the reference standard may however show selection bias by exclusion of men with negative biopsy or patients not suitable for RP. In our study 91 patients (37.1%) treated with radiation therapy were included although the diagnosis in these patients relies solely on prostate biopsy. It may be estimated that about 20% of these patients who were treated for a low-grade PC might have harbored high-grade PC that remained undetected [26]. Also, in the patients that were considered free of cancer there is no perfect way to prove the absence with 100 % certainty. We considered a minimum of two years of follow-up as a reasonable time for a life-threatening ClinsigPC that might have been present at time of the MRI to appear. Secondly, our histopathological and clinical follow-up reference was determined on a per-patient basis, thus correlation of the PI-RADS scores with regional location of the PC in the prostate was not possible. We have chosen to evaluate the scoring systems in the most relevant setting for clinicians in daily practice because the overall assessment score will eventually be communicated in the report as a simplified risk-stratification system providing recommendations for further diagnostic procedures. Thirdly, the PIRADSv1 and PIRADSv2 scores were assigned by a single reader during the same reading session, and not in a blinded manner. This may have induced homogenization of the scores, but was necessary for the purpose of the study, which was to compare the scores, not to evaluate the reader.

We did not compare the inter- and intra-observer variability of PI-RADSv1 and PI-RADSv2 in the current study, but this may be an interesting topic for future research. A last limitation may be the definition used for ClinsigPC in our study. Unfortunately, there is currently a lack of consensus among urologists about what constitutes clinically significant disease, therefore we decided to use the definition as proposed in PI-RADSv2 [5], although we are aware that other definitions might have resulted in different performance characteristics.

## Conclusion

The mpMRI scoring systems PI-RADSv1 and PI-RADSv2 yield similar accuracy to detect ClinsigPC in patients with elevated PSA, although clinicians should be aware that when an overall assessment score of 3 is used as a threshold for a positive mpMRI, PI-RADSv2 has lower sensitivity than PI-RADSv1. Nevertheless, PI-RADSv2 is preferable over PI-RADSv1 because it has the advantage of providing well defined instructions on how to determine the overall assessment category.

## Supplementary Files

The supplementary files for this article can be found as follows:

- **Supplementary File 1: Table 1. **jbsr-100-1-1147-s1.pdf
  DOI: http://dx.doi.org/10.5334/jbsr.1147.s1
- **Supplementary File 2: Table 2. **jbsr-100-1-1147-s2.pdf
  DOI: http://dx.doi.org/10.5334/jbsr.1147.s2
- **Supplementary File 3: Table 3. **jbsr-100-1-1147-s3.pdf
  DOI: http://dx.doi.org/10.5334/jbsr.1147.s3
