# Supplementary material for: Comparison of the Prostate Imaging Reporting and Data System (PI-RADS) Version 1 and 2 in a Cohort of 245 Patients with Histopathological Reference and Long-Term Follow-Up
Source: J Belg Soc Radiol. 2016 Nov 24;100(1):108. doi: 10.5334/jbr-btr.1147 (PMC5854270; doi:10.5334/jbr-btr.1147)
Supplement: Supplementary file 1 [file jbsr-100-1-1147-s1.pdf]

Table 1: mpMRI technical parameters

3.0T Magnetom Trio, Siemens, Erlangen, Germany		TR (msec)	TE (msec)	Flip angle	Plane	number of slices	voxel size (mm)	matrix size (mm)	FOV (mm)
T2-WI	Turbo Spin Echo	8000	96	120°	axial	20	0.4 x 0.4 x 3.0	410 x 512	300 x 300
DWI	Single-Shot Echo-Planar Imaging with Fat suppression<br>b values 50, 250, 500, 750, 1000 sec/mm²	2500	77	NA	axial	20	1.5 x 1.5 x 5.0	115 x 192	285 x 285
MRSI	CSI point-resolved spatially localized spectroscopy (PRESS), FWHM 8 Hz	750	145	90°	3D	NA	4.0 x 4.0 x 5.0 (nominal voxel size 6mm³)	NA	300 x 300
DCE	T1-3D-GRE (VIBE), 36 series every 9 sec after manual IV injection of 0,2 ml/kg body weight Gd-DTPA-BMA (Omniscan, GE Healthcare, Diegem, Belgium) followed by a 15 ml saline flush	4.9	1.7	10°	axial	20	1.5 x 1.5 x 3.6	138 x 192	260 x 260
