# Supplementary material for: Comparison of the Prostate Imaging Reporting and Data System (PI-RADS) Version 1 and 2 in a Cohort of 245 Patients with Histopathological Reference and Long-Term Follow-Up
Source: J Belg Soc Radiol. 2016 Nov 24;100(1):108. doi: 10.5334/jbr-btr.1147 (PMC5854270; doi:10.5334/jbr-btr.1147)
Supplement: Supplementary file 2 [file jbsr-100-1-1147-s2.pdf]

Supplemental table 2: The PI-RADSv1 scoring system, adapted from Barentsz et al. [1]

T2WI for the peripheral zone (PZ)
1: Uniform high signal intensity (SI)
2: Linear, wedge shaped, or geographic areas of lower SI, usually not well demarcated
3: Intermediate appearances not in categories 1/2 or 4/5
4: Discrete, homogeneous low signal focus/mass confined to the prostate
5: Discrete, homogeneous low signal intensity focus with extra-capsular extension/invasive behaviour or mass effect on the capsule (bulging), or broad (>1.5 cm) contact with the surface

T2WI for the transition zone (TZ)
1: Heterogeneous TZ adenoma with well-defined margins: "organised chaos"
2: Areas of more homogeneous low SI, however well marginated, originating from the TZ/BPH
3: Intermediate appearances not in categories 1/2 or 4/5
4: Areas of more homogeneous low SI, ill defined: "erased charcoal sign"
5: Same as 4, but involving the anterior fibromuscular stroma or the anterior horn of the PZ, usually lenticular or water-drop shaped.

Diffusion weighted imaging (DWI)
1: No reduction in ADC compared with normal glandular tissue. No increase in SI on any high b-value image (≥b800)
2: Diffuse, hyper SI on ≥b800 image with low ADC; no focal features, however, linear, triangular or geographical features are allowed
3: Intermediate appearances not in categories 1/2 or 4/5
4: Focal area(s) of reduced ADC but iso-intense SI on high b-value images (≥b800)
5: Focal area/mass of hyper SI on the high b-value images (≥b800) with reduced ADC

Dynamic contrast enhanced (DCE)-MRI
1: Type 1 enhancement curve
2: Type 2 enhancement curve
3: Type 3 enhancement curve
+1: For focal enhancing lesion with curve type 2–3
+1: For asymmetric lesion or lesion at an unusual place with curve type 2–3

Spectroscopy (MRSI)
In at least three adjacent voxels:
1: Citrate peak height exceeds choline peak height >2 times
2: Citrate peak height exceeds choline peak height times >1, <2 times
3: Choline peak height equals citrate peak height
4: Choline peak height exceeds citrate peak height >1, <2 times
5: Choline peak height exceeds citrate peak height >2 times

PI-RADSv1 overall assessment category: subjective impression based on weighting of all modalities
Score 1: Clinically significant disease is highly unlikely to be present
Score 2: Clinically significant cancer is unlikely to be present
Score 3: Clinically significant cancer is equivocal
Score 4: Clinically significant cancer is likely to be present
Score 5: Clinically significant cancer is highly likely to be present.
