# Supplementary material for: Comparison of the Prostate Imaging Reporting and Data System (PI-RADS) Version 1 and 2 in a Cohort of 245 Patients with Histopathological Reference and Long-Term Follow-Up
Source: J Belg Soc Radiol. 2016 Nov 24;100(1):108. doi: 10.5334/jbr-btr.1147 (PMC5854270; doi:10.5334/jbr-btr.1147)
Supplement: Supplementary file 3 [file jbsr-100-1-1147-s3.pdf]

Supplemental table 3: PI-RADSv2 scoring system, adapted from Weinreb et al. [4]

T2 for the peripheral zone (PZ)

1: Uniform hyperintense SI (normal)
2: Linear or wedge-shaped hypointensity or diffuse mild hypointensity, usually indistinct margin
3: heterogeneous SI or non-circumscribed, rounded, moderate hypointensity. Includes others that do not qualify as 2, 4 or 5
4: Circumscribed, homogenous moderate hypointense focus/mass, confined to prostate and <1,5cm
5: Same as 4, but ≥1.5 cm or definite EPE/invasive behaviour

T2 for the transition zone (TZ)

1: homogeneous intermediate SI (normal)
2: circumscribed hypointense or heterogeneous encapsulated nodule(s) (BPH)
3: heterogeneous SI with obscured margins, includes others that do not qualify as 2, 4 or 5
4: lenticular or non-circumscribed, homogenous moderately hypointense, and <1,5cm
5: Same as 4, but ≥1.5 cm or definite EPE/invasive behaviour

Diffusion weighted imaging (DWI)

1: No abnormality. High SI on ADC and low SI on high b-value image (≥b800)
2: indistinct hypointense on ADC
3: focal mildly/moderately hypointense on ADC and isointense/mildly hyperintense on high b-value DWI
4: focal markedly hypointense on ADC and markedly hyperintense on high b-value; and <1,5cm
5: idem as 4, but ≥1.5 cm or definite EPE/invasive behaviour

Dynamic contrast enhanced (DCE)-MRI

Negative:
- No enhancement
- Or diffuse enhancement not corresponding to a focal finding on T2 and/or DWI
- Or focal enhancement corresponding to BPH nodule on T2

Positive:
- Focal enhancement
- And earlier than or contemporaneously with enhancement adjacent normal prostatic tissue
- And corresponds to suspicious finding on T2 and/or DWI

PI-RADSv2 overall assessment category: DWI dominant modality in PZ, T2 dominant modality in TZ

PERIPHERAL ZONE

DWI	T2	DCE	PI-RADS score
1	Any	Any	1
2	Any	Any	2
3	Any	Negative	3
		Positive	4
4	Any	Any	4
5	Any	Any	5

TRANSITION ZONE

DWI	T2	DCE	PI-RADS score
Any	1	Any	1
Any	2	Any	2
1-4	3	Any	3
5			4
Any	4	Any	4
Any	5	Any	5
